# Structured Sanda training as an innovative exercise intervention for young men with obesity: metabolic load distribution and adaptive responses compared with moderate-intensity continuous cycling

**DOI:** 10.3389/fspor.2026.1916278

**Published:** 2026-08-10

**Authors:** Aijiao Chen, Xin Tian, Mengdi Tian

**Affiliations:** 1 Chengdu Sport University, Chengdu, China; 2 Sichuan Technology and Business University, Sichuan, China

**Keywords:** cardiorespiratory fitness, combat-sport-based exercise, fat oxidation, metabolic load, moderate-intensity continuous training, obesity, Sanda training, ventilatory threshold

## Abstract

**Background:**

Innovative and engaging exercise interventions are needed to improve cardiometabolic health and exercise adherence in young adults with obesity. Sanda, a Chinese combat sport characterized by footwork, striking combinations, defensive movements, and attack–recovery transitions, may provide a practical intermittent exercise modality when delivered in a structured and low-contact format. However, its metabolic-load characteristics and adaptive effects in individuals with obesity remain insufficiently understood.

**Objective:**

This study compared the effects of 12 weeks of structured low-contact Sanda training and moderate-intensity continuous cycling training on body composition, cardiorespiratory fitness, ventilatory thresholds, and substrate oxidation profiles in young men with obesity, while describing the real-time metabolic-load distribution of both exercise modalities.

**Methods:**

Thirty young men with obesity were randomly assigned to a structured Sanda training group or a moderate-intensity continuous cycling group. Both groups completed a 12-week intervention consisting of three 60-min sessions per week. Body composition was assessed before and after the intervention. A graded incremental cycling test was used to determine peak oxygen uptake, first and second ventilatory thresholds, maximal fat oxidation, and FATmax. During training sessions, oxygen uptake, carbon dioxide production, heart rate, respiratory exchange ratio, and energy expenditure were continuously monitored using a portable metabolic system.

**Results:**

Both interventions significantly improved body mass, body mass index, body fat percentage, cardiorespiratory fitness, and substrate oxidation indicators. Compared with moderate-intensity continuous cycling, structured Sanda training produced greater improvements in peak oxygen uptake and second ventilatory threshold power, whereas cycling training produced larger improvements in body fat percentage, maximal fat oxidation, and FATmax. Although total session energy expenditure was broadly comparable between groups, Sanda training showed a more intermittent metabolic-load pattern, with higher peak oxygen uptake, higher peak heart rate, and a greater proportion of time with respiratory exchange ratio above 0.95. In contrast, cycling training showed a more stable moderate-intensity load distribution.

**Conclusion:**

Under equal-duration intervention conditions, structured Sanda training and moderate-intensity continuous cycling both improved cardiometabolic and fitness-related outcomes in young men with obesity, but their adaptive profiles differed. Structured low-contact Sanda training may serve as an engaging combat-sport-based exercise option for improving cardiorespiratory fitness and high-intensity exercise tolerance, whereas moderate-intensity continuous cycling may be more favorable for improving fat oxidation profiles and reducing body fat. These findings support the potential value of structured Sanda training as an innovative exercise intervention for obesity-related health promotion, while highlighting the need to interpret the results as a comparison between two practical exercise modalities rather than as a fully load-matched comparison between standardized HIIT and MICT protocols.

## 1 Introduction

Obesity has become one of the most serious public health challenges worldwide and is associated with substantially increased risks of cardiovascular disease, type 2 diabetes, metabolic syndrome, and premature mortality (1). Beyond excessive adiposity, obesity is characterized by reduced insulin sensitivity, dysregulated lipid metabolism, impaired cardiorespiratory fitness (CRF), and abnormalities in substrate utilization (2–4). Among these factors, CRF has emerged as one of the strongest independent predictors of cardiovascular health and all-cause mortality, highlighting the importance of exercise-based interventions for improving both physiological function and long-term health outcomes in individuals with obesity.

Exercise-induced substrate utilization is an important component of metabolic health. The ability to oxidize fat during exercise is commonly evaluated using maximal fat oxidation (MFO) and the exercise intensity eliciting MFO (FATmax), which are derived from respiratory gas-exchange measurements (5, 6). Reduced fat oxidation capacity has been linked to impaired metabolic flexibility, a concept describing the ability of the body to appropriately switch between carbohydrate and lipid utilization according to changing metabolic demands and substrate availability (7, 8). Although MFO and FATmax do not directly quantify whole-body metabolic flexibility, they provide valuable indicators of exercise-related fat oxidation capacity and substrate oxidation profiles and are therefore widely used in obesity and exercise metabolism research.

Exercise training is considered a cornerstone strategy for obesity prevention and treatment. Moderate-intensity continuous training (MICT) remains one of the most widely recommended exercise approaches because of its safety, controllability, and reproducibility (9, 10). By maintaining exercise within a relatively stable aerobic intensity range, MICT promotes energy expenditure, enhances fat oxidation, and contributes to improvements in body composition and cardiometabolic health (11). In recent years, high-intensity interval training (HIIT) has attracted considerable attention because it can induce substantial improvements in cardiorespiratory fitness and cardiometabolic function within relatively short training durations (12–16). Previous studies have suggested that MICT and HIIT may produce different physiological adaptations owing to their distinct intensity distributions and metabolic demands, with HIIT generally eliciting greater improvements in cardiorespiratory fitness and higher-intensity exercise capacity, whereas MICT may provide advantages for sustained fat oxidation and long-duration aerobic metabolism.

Despite their suggested effectiveness, both MICT and conventional laboratory-based HIIT protocols present practical limitations. Continuous cycling or treadmill exercise may be perceived as monotonous by some individuals, potentially reducing long-term exercise adherence (17). Conversely, standardized HIIT protocols often require strict intensity control, specialized equipment, and substantial motivation, which may limit their implementation in real-world settings. In addition, affective responses during exercise may influence future motivation and physical activity behavior, suggesting that the subjective experience of exercise is important for long-term participation (18). Therefore, increasing attention has been directed toward alternative exercise modalities that combine physiological effectiveness with greater enjoyment, engagement, and long-term adherence.

Combat-sport-based exercise has emerged as a promising alternative form of structured physical activity. Compared with traditional endurance exercise, combat-sport activities typically involve whole-body movement patterns, rapid directional changes, repeated transitions between high- and low-intensity actions, and greater technical and cognitive engagement. These characteristics may generate unique physiological stimuli while simultaneously enhancing exercise enjoyment and participation. Previous studies involving martial arts, boxing, taekwondo, karate, and other combat-sport modalities have reported improvements in aerobic fitness, body composition, metabolic health, and health-related physical fitness (19–24). Such findings suggest that combat-sport-based exercise may represent a practical and attractive alternative to more traditional exercise prescriptions.

Sanda is a Chinese combat sport that integrates footwork, punching and kicking combinations, defensive maneuvers, and repeated attack–recovery transitions. From both biomechanical and physiological perspectives, Sanda represents a complex whole-body activity involving multi-joint movement patterns, intermittent high-intensity actions, and frequent fluctuations in metabolic demand. In addition to its physical demands, Sanda requires technical execution, motor coordination, rapid decision-making, and continuous engagement, characteristics that may enhance exercise enjoyment and long-term participation. Previous Sanda-related studies have examined sport-specific kicking mechanics, high-intensity conditioning protocols, and cognitive–motor control characteristics in Sanda practitioners, suggesting that Sanda involves both demanding physical actions and complex neuromotor engagement (25–27).

Importantly, Sanda training can be delivered in a structured low-contact format emphasizing technical drills, pad work, movement combinations, and controlled attack–recovery exercises rather than competitive sparring or full-contact combat. Such an approach may reduce injury risk while preserving the dynamic and intermittent characteristics of the sport. Physiologically, repeated offensive and defensive actions may induce fluctuations in oxygen uptake, ventilation, heart rate, and substrate utilization that resemble the intensity oscillations observed during interval training. Nevertheless, whether structured Sanda training should be considered equivalent to standardized HIIT remains uncertain because objective verification of exercise intensity relative to ventilatory thresholds is rarely available. Therefore, in the present study, Sanda is considered an intermittent combat-sport-based exercise modality with HIIT-like characteristics rather than a formal HIIT protocol.

Although combat-sport-based exercise has suggested potential benefits for health and fitness, considerably less is known about the metabolic-load characteristics of structured Sanda training in individuals with obesity. Existing studies have primarily focused on sport performance, physical fitness, technique execution, or cognitive–motor characteristics. However, real-time metabolic responses during structured Sanda training remain poorly understood. Furthermore, although portable respiratory metabolic systems have been validated for field-based physiological monitoring (28), few investigations have employed such systems to characterize the temporal distribution of metabolic load during Sanda training sessions. Consequently, it remains unclear whether structured Sanda training produces adaptive responses that differ from those induced by conventional MICT under comparable intervention durations. Therefore, the novelty of the present study lies not in re-establishing the relative superiority of interval vs. continuous exercise, but rather in characterizing the metabolic-load distribution of structured Sanda training and examining its associated physiological adaptations.

Therefore, this study employed a 12-week randomized controlled intervention to compare structured low-contact Sanda training with cycling-based MICT in young men with obesity. The primary aim was to compare the effects of the two exercise modalities on body composition, cardiorespiratory fitness, ventilatory thresholds, and substrate oxidation profiles while characterizing their metabolic-load distributions using continuous respiratory monitoring. We hypothesized that both interventions would improve health-related outcomes, but that differences in metabolic-load distribution would lead to distinct physiological adaptations.

## 2 Participants and methods

### 2.1 Study design

**Figure 1:**
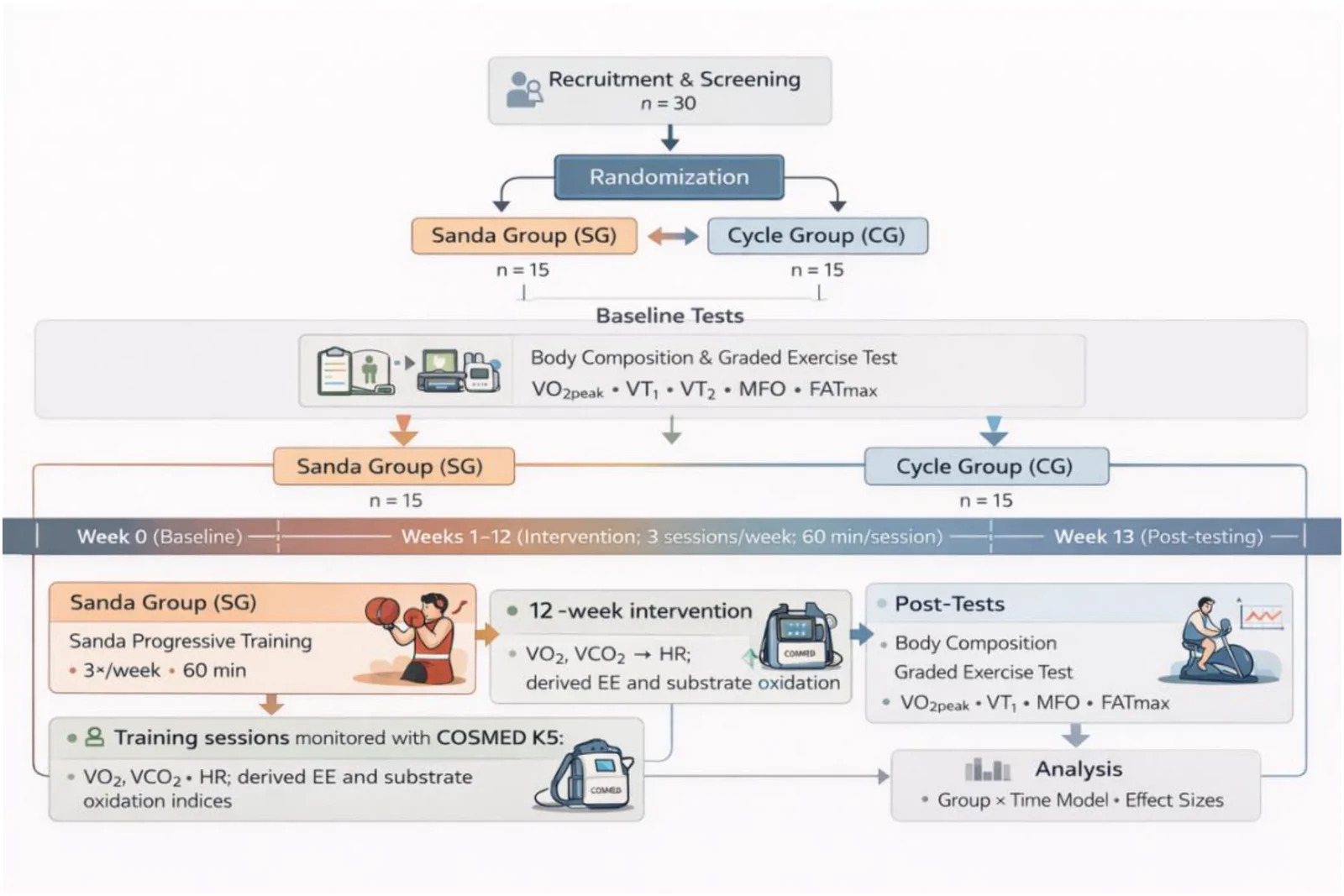
Study protocol. The diagram was created by the authors using Microsoft PowerPoint and manually revised for publication. No generative AI tool was used to generate the scientific content of the figure.

This study was a 12-week randomized parallel-group intervention study. Thirty young men with obesity who met the eligibility criteria were randomly assigned to either the structured Sanda training group (SG) or the moderate-intensity continuous cycling group (CG) using a computer-generated randomization sequence. Because of the nature of the exercise intervention, blinding of participants and exercise instructors was not feasible. However, all physiological measurements were performed according to standardized protocols, and data processing followed predefined analytical procedures. Both groups completed equal-duration exercise interventions (3 sessions/week, 60 min/session) over 12 weeks. Although the two interventions were matched for session duration and training frequency, they were not intended to produce identical physiological training stimuli because of the inherent differences between exercise modalities. Body composition and graded incremental cycling tests were performed before and after the intervention. The main outcomes were V̇O_2_peak, VT1, VT2, MFO, and FATmax. During the intervention, participants wore a COSMED K5 portable metabolic system during training sessions to record V̇O_2_, V̇CO_2_, HR, RER, and energy expenditure, allowing the metabolic-load distribution and integrated physiological demands of each exercise modality to be characterized throughout the intervention (Figure 1).

### 2.2 Participants

A total of 30 young male participants with obesity were recruited. The inclusion criteria were: (1) age 18–35 years; (2) BMI ≥ 28 kg/m^2^; (3) no regular physical training in the past 3 months; (4) no contraindications to exercise due to cardiovascular, respiratory, or musculoskeletal conditions; and (5) provision of written informed consent. Obesity was defined as BMI ≥ 28 kg/m^2^ according to the Chinese adult BMI classification criteria. Because this criterion differs from the WHO obesity definition (BMI ≥ 30 kg/m^2^), caution is warranted when comparing the present findings with studies using the WHO classification. The exclusion criteria included smoking, habitual heavy alcohol consumption, use of medications known to affect metabolism or exercise performance (e.g., glucocorticoids, β-blockers, hypoglycemic agents, or anti-obesity medications), inability to complete the tests or training, or adverse events affecting continued participation. The study protocol was approved by the Ethics Review Committee of Chengdu Sport University (approval No. 2026104). All participants provided written informed consent before enrollment.

### 2.3 Experimental control

Tests were scheduled at fixed time points in the morning. Participants were instructed to avoid strenuous exercise 24 h before testing and to abstain from alcohol and excessive caffeine intake for at least 48 h before testing. The tests were conducted with participants fasting from 10 to 12 h on the test day (only water was allowed). The laboratory temperature was maintained at 24 °C–26 °C with 40%–60% humidity.

### 2.4 Dietary control

Participants were not prescribed a weight-loss diet and were not provided standardized meals. To minimize diet-related confounding, they were instructed to maintain their habitual dietary patterns throughout the intervention and to avoid intentional dieting, binge eating, or major changes in macronutrient intake. General dietary guidance was provided only to support stability of habitual intake rather than to induce dietary weight loss. This guidance included maintaining regular meal timing, avoiding intentional caloric restriction, avoiding binge eating, and maintaining habitual macronutrient intake throughout the intervention. Brief food diaries or 24-h dietary recalls were collected at baseline and during the intervention to check whether participants reported major dietary changes. When large deviations from habitual intake were identified, participants were reminded to return to their usual eating pattern. Because total energy intake and macronutrient intake were not quantitatively analyzed as intervention outcomes, diet-related influences cannot be fully excluded and are acknowledged as a limitation.

### 2.5 Exercise intervention protocol

#### 2.5.1 Sanda training group (SG)

The SG completed a structured, supervised, low-contact Sanda training program for 12 weeks (3 sessions/week, 60 min/session). Each session consisted of a 10-min warm-up, 40-min main training period, and 10-min cool-down. The intervention emphasized footwork, punching and kicking combinations, pad work, defensive movements, and controlled attack-recovery drills. Full-contact sparring and competitive throwing were not used, and all sessions were supervised by qualified instructors to reduce injury risk.

Weeks 1–4 (adaptation phase): basic stance, footwork, shadow drills, and low-to-moderate-intensity pad practice; target intensity 60%–75% HRmax.

Weeks 5–8 (development phase): technical combinations and pad-work intervals, typically 6–8 sets of 45 s attack actions followed by 45 s active recovery; target intensity 70%–85% HRmax.

Weeks 9–12 (consolidation phase): longer combination drills and pad-work intervals, typically 8–10 sets of 60 s high-intensity actions followed by 60 s low-intensity movement recovery; peak intensity was allowed to reach approximately 85%–95% HRmax. Because VT-zone verification during each action segment was not used as an inclusion criterion, this intervention is referred to as HIIT-like intermittent Sanda training rather than standardized HIIT.

#### 2.5.2 Moderate-intensity continuous cycling group (CG)

The CG completed cycling MICT for 12 weeks (3 sessions/week, 60 min/session), including 10 min warm-up, 40 min main cycling exercise, and 10 min cool-down. The main training intensity was controlled at 65%–75% HRmax or near VT1, with cadence and power adjusted to maintain a stable moderate-intensity workload.

Both groups wore the COSMED K5 system and a heart rate monitor during training sessions to record heart rate (HR), Rating of Perceived Exertion (RPE), training attendance, and adverse events. The Sanda group used low-contact drills and pad work only; no full-contact sparring was performed during the intervention. Adherence was assessed as the percentage of scheduled sessions completed. For the per-protocol analysis, participants were required to complete post-intervention testing and attend at least 85% of the scheduled training sessions. All training sessions in both groups were supervised by experienced exercise instructors and implemented according to standardized procedures to ensure protocol fidelity and consistency throughout the intervention period.

### 2.6 Graded incremental cycling test

The graded incremental cycling test was performed at 60 rpm (±5). After a 3-min warm-up at 0–20 W, workload increased by 20 W every 3 min starting from 30 W until volitional exhaustion. V̇O_2_peak was determined as the highest 30-s average V̇O_2_ value during the final stage. VT1 and VT2 were identified using the V-slope method combined with ventilatory equivalents. VT1 was defined as the first nonlinear increase in V̇CO_2_ relative to V̇O_2_, accompanied by an increase in V̇E/V̇O_2_ without a concomitant increase in V̇E/V̇CO_2_. VT2 was defined as the second ventilatory breakpoint, characterized by a systematic increase in V̇E/V̇CO_2_, a further rise in V̇E/V̇O_2_, and a clear hyperventilatory response. Two trained researchers independently identified VT1 and VT2; disagreements were resolved by discussion with a third researcher. For substrate oxidation calculations (29), V̇O_2_ and V̇CO_2_ during the last 60 s of each stage were used, and only stages with RER ≤ 1.00 were included in MFO/FATmax calculations.

### 2.7 Substrate oxidation and energy expenditure calculation (according to established formulas)

V̇O_2_ and V̇CO_2_ are expressed in L/min:

$$\mathrm{EE}\;(\mathrm{kcal}/\min)=3.716\times \dot{V}O_{2}+1.332\times \dot{V}{\mathrm{CO}}_{2}$$

$$\mathrm{CHOox}\;(g/\min)=4.585\times \dot{V}{\mathrm{CO}}_{2}-3.2256\times \dot{V}O_{2}$$

$$\mathrm{FATox}(g/\min)=1.695\times \dot{V}O_{2}-1.701\times \dot{V}{\mathrm{CO}}_{2}$$

MFO is the maximum value of FATox during the graded test; FATmax is the corresponding power at that point (30).

### 2.8 Continuous monitoring during training and summary of indicators

In the present study, metabolic load was operationally defined as the integrated physiological demand imposed during exercise, characterized by oxygen uptake (VO_2_), carbon dioxide production (VCO_2_), respiratory exchange ratio (RER), heart rate (HR), and energy expenditure (EE). These variables were selected because they collectively reflect the cardiorespiratory and metabolic responses to exercise rather than representing a single physiological construct. During each training session, both groups wore the COSMED K5 portable metabolic analysis system for continuous monitoring, collecting time-series data for V̇O_2_, V̇CO_2_, RER, energy expenditure (EE), and heart rate (HR). Raw respiratory metabolic data were collected breath-by-breath and smoothed using moving averages to minimize the impact of instantaneous fluctuations.

For each training session, total energy expenditure (kcal/session), average and peak V̇O_2_, average and peak HR, average RER, and the proportion of time with RER > 0.95 were summarized. These variables were used to characterize the metabolic-load distribution rather than to claim full equivalence of physiological or biomechanical load between modalities. Equal intervention duration and broadly comparable total session energy expenditure were used only to standardize the intervention protocol and should not be interpreted as indicating equivalent physiological or biomechanical training stimuli between the two exercise modalities. Additionally, representative training sessions were selected to plot the EE (kcal/min) over time, visually demonstrating the differences in metabolic load distribution between the training modes.

### 2.9 Statistical analysis

Sample size estimation was conducted using G*Power 3.1.9.7 software. Based on the repeated measures analysis of variance (ANOVA: repeated measures, within–between interaction), effect size f = 0.25, significance level *α* = 0.05, power = 0.80, number of groups = 2, and measurement times = 2, and using a pre-study and similar research estimate of the correlation between repeated measures (*ε* = 1), the minimum total sample size required was approximately 28 participants. Considering potential dropouts and missing data, a total of 30 participants were included.

Statistical analysis was performed using SPSS software. Continuous variables were presented as mean ± standard deviation. The normality of the data was assessed using the Shapiro–Wilk test. Baseline data were reported according to the randomized group allocation. The primary outcome analysis before and after the intervention used a per-protocol analysis, defined as including participants who completed post-intervention testing and attended at least 85% of scheduled training sessions. A 2 (group: SG/CG) × 2 (time: pre-intervention/post-intervention) repeated measures ANOVA was performed to test for main effects of group, time, and group × time interaction. For significant interaction effects, simple effects analysis was conducted, and multiple comparisons were corrected using the Bonferroni method. Results were reported with *P*-values and effect sizes (partial eta squared). Partial eta squared values were interpreted as small (≥0.01), moderate (≥0.06), and large (≥0.14). All tests were two-sided, with statistical significance set at *P* < 0.05.

All analyses were performed on a per-protocol basis because one participant in the SG did not complete the post-intervention assessment.

## 3 Results

### 3.1 Participant completion and baseline characteristics

A total of 30 participants were recruited and randomly assigned to either the Sanda training group (SG = 15) or the moderate-intensity continuous cycling group (CG = 15). During the intervention, one participant in SG did not complete the post-intervention testing and was excluded from the per-protocol analysis. The remaining participants completed post-intervention testing and met the predefined adherence criterion of attending at least 85% of scheduled training sessions. Therefore, the per-protocol analysis included 14 participants in SG and 15 in CG. Baseline characteristics are shown in Table 1.

**Table 1:** Baseline characteristics of participants (mean ± SD).

Variable	SG (*n* = 15)	CG (*n* = 15)
Age (years)	24.3 ± 3.5	23.7 ± 3.9
Height (cm)	174.2 ± 6.1	175.8 ± 5.4
Body mass (kg)	89.6 ± 9.0	92.3 ± 8.7
BMI (kg·m⁻^2^)	29.3 ± 2.0	29.9 ± 1.7
Body fat (%)	30.9 ± 2.9	31.8 ± 2.4
V̇O_2_peak (mL·kg⁻^1^·min⁻^1^)	27.6 ± 4.2	28.5 ± 3.8
VT1 power (W)	90 ± 16	96 ± 14
VT2 power (W)	128 ± 20	133 ± 18
MFO (g·min⁻^1^)	0.27 ± 0.08	0.30 ± 0.07
FATmax (W)	78 ± 15	84 ± 14

SG, Sanda training group; CG, moderate-intensity continuous cycling training group; BMI, body mass index; V̇O_2_peak, peak oxygen uptake; VT1/VT2, first and second ventilatory thresholds; MFO, maximal fat oxidation rate; FATmax, exercise intensity eliciting MFO.

One participant in the SG did not complete the post-intervention assessment and was therefore excluded from the per-protocol analysis. All remaining participants completed the intervention and post-intervention testing.

### 3.2 Changes in body composition

Changes in body composition and the results of the repeated-measures ANOVA are presented in Table 2. Body mass decreased by 2.2 kg in SG and 3.8 kg in CG, and BMI decreased by 0.7 kg/m^2^ in SG and 1.2 kg/m^2^ in CG. Significant main effects of time were observed for both body mass and BMI (*P* for time effect < 0.001), whereas group × time interactions were not significant (body mass: *P* for interaction = 0.100; BMI: *P* for interaction = 0.120). Body fat percentage decreased by 1.8 percentage points in SG and 3.1 percentage points in CG, with a significant group × time interaction (*P* for interaction = 0.040, partial eta squared = 0.147), suggesting a larger reduction in CG.

**Table 2:** Changes in body composition and results of repeated-measures ANOVA (mean ± SD).

Variable	SG Pre	SG Post	CG Pre	CG Post	*P* group	*P* time	*P* interaction	*ηp*^2^ interaction
Body mass (kg)	89.6 ± 9.0	87.4 ± 8.8	92.3 ± 8.7	88.5 ± 8.6	0.190	<0.001	0.100	0.097
BMI (kg·m⁻^2^)	29.3 ± 2.0	28.6 ± 1.9	29.9 ± 1.7	28.7 ± 1.7	0.150	<0.001	0.120	0.087
Body fat (%)	30.9 ± 2.9	29.1 ± 2.8	31.8 ± 2.4	28.7 ± 2.9	0.210	<0.001	0.040	0.147

Per-protocol analysis: SG *n* = 14, CG *n* = 15. A two-way repeated-measures ANOVA [group (SG vs. CG) × time (pre vs. post)] was performed. *P* group, *P* time, and *P* interaction represent the main effects of group, time, and their interaction, respectively. Partial eta squared (*ηp*^2^) is reported for interaction effects only.

### 3.3 Cardiorespiratory fitness and ventilatory thresholds

Changes in cardiorespiratory fitness and ventilatory threshold parameters are presented in Table 3. V̇O_2_peak increased by 4.4 mL·kg⁻^1^·min⁻^1^ in SG and 1.6 mL·kg⁻^1^·min⁻^1^ in CG. VT2 power increased by 27 W in SG and 12 W in CG. Both V̇O_2_peak and VT2 power showed significant main effects of time (*P* for time effect < 0.001) and significant group × time interactions (V̇O_2_peak: *P* for interaction = 0.007, partial eta squared = 0.240; VT2: *P* for interaction = 0.002, partial eta squared = 0.302), indicating larger increases in SG. VT1 power increased by 18 W in SG and 11 W in CG, with a significant main effect of time (*P* for time effect < 0.001) but no significant interaction (*P* for interaction = 0.180).

**Table 3:** Changes in cardiorespiratory fitness and ventilatory thresholds (mean ± SD).

Variable	SG Pre	SG Post	CG Pre	CG Post	*P* group	*P* time	*P* interaction	*ηp*^2^ interaction
V̇O_2_peak (mL·kg⁻^1^·min⁻^1^)	27.6 ± 4.2	32.0 ± 4.6	28.5 ± 3.8	30.1 ± 4.0	0.620	<0.001	0.007	0.240
VT1 power (W)	90 ± 16	108 ± 17	96 ± 14	107 ± 16	0.380	<0.001	0.180	0.066
VT2 power (W)	128 ± 20	155 ± 22	133 ± 18	145 ± 19	0.410	<0.001	0.002	0.302

Per-protocol analysis: SG *n* = 14, CG *n* = 15. A two-way repeated-measures ANOVA [group (SG vs. CG) × time (pre vs. post)] was performed. *P* group, *P* time, and *P* interaction represent the main effects of group, time, and their interaction, respectively. Partial eta squared (*ηp*^2^) is reported for interaction effects only.

### 3.4 Substrate oxidation responses

Changes in substrate oxidation parameters are summarized in Table 4. MFO increased by 0.05 g/min in SG and 0.08 g/min in CG, while FATmax increased by 10 W in SG and 19 W in CG. Both MFO and FATmax showed significant main effects of time (*P* for time effect < 0.001) and significant group × time interactions (MFO: *P* for interaction = 0.030, partial eta squared = 0.163; FATmax: *P* for interaction = 0.020, partial eta squared = 0.185), indicating larger improvements in CG. RER at 100 W decreased slightly in both groups, with a significant main effect of time (*P* for time effect = 0.010) but no significant interaction (*P* for interaction = 0.310).

**Table 4:** Changes in substrate oxidation responses (mean ± SD) per-protocol analysis: SG *n* = 14, CG *n* = 15.

Variable	SG Pre	SG Post	CG Pre	CG Post	*P* group	*P* time	*P* interaction	*ηp*^2^ interaction
MFO (g·min⁻^1^)	0.27 ± 0.08	0.32 ± 0.09	0.30 ± 0.07	0.38 ± 0.10	0.170	<0.001	0.030	0.163
FATmax (W)	78 ± 15	88 ± 16	84 ± 14	103 ± 17	0.090	<0.001	0.020	0.185
RER@100W	0.95 ± 0.05	0.94 ± 0.06	0.96 ± 0.05	0.93 ± 0.06	0.480	0.010	0.310	0.038

A two-way repeated-measures ANOVA [group (SG vs. CG) × time (pre vs. post)] was performed. *P* group, *P* time, and *P* interaction represent the main effects of group, time, and their interaction, respectively. Partial eta squared (*ηp*^2^) is reported for interaction effects only. RER, respiratory exchange ratio measured at a fixed power of 100 W.

### 3.5 Summary of continuous training monitoring

Summary metrics for individual sessions are presented in Table 5. Total energy expenditure per session was similar between groups (SG: 540 ± 120 kcal; CG: 520 ± 85 kcal). This indicates that the overall session dose was broadly comparable in duration and total energy output, but it should not be interpreted as evidence that the two modalities were matched for physiological or biomechanical load.

**Table 5:** Summary of continuous training monitoring (mean ± SD per session).

Variable	SG (Sanda)	CG (MICT, cycle ergometer)
Total EE per session (kcal)	540 ± 120	520 ± 85
Average V̇O_2_ (L·min⁻^1^)	1.62 ± 0.28	1.55 ± 0.22
Peak V̇O_2_ (L·min⁻^1^)	2.85 ± 0.45	2.10 ± 0.30
Average HR (bpm)	148 ± 12	142 ± 10
Peak HR (bpm)	184 ± 9	164 ± 10
Average RER	0.93 ± 0.04	0.90 ± 0.03
Time with RER > 0.95 (%)	34 ± 12	18 ± 9

All training sessions were recorded using the COSMED K5 system. Individual session metrics (total EE, average and peak V̇O_2_ and HR, etc.) were first summarized, and then the mean ± SD across all valid sessions was calculated for each group. EE, energy expenditure; HR, heart rate; RER, respiratory exchange ratio.

**Figure 2:**
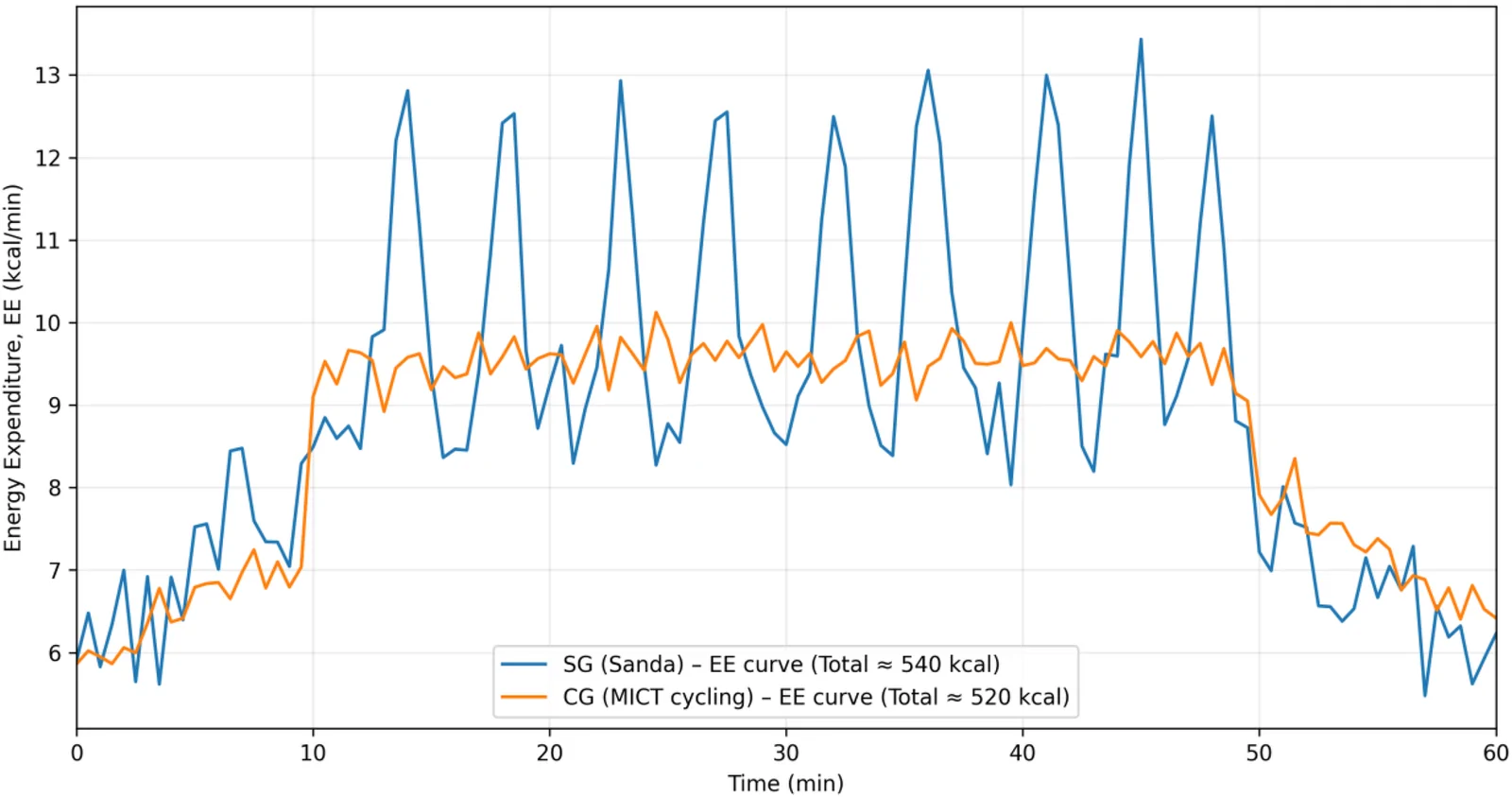
Representative energy expenditure profiles during a 60-min training session.

The time-course of energy expenditure rate (EE, kcal/min) for a representative training session is shown in Figure 2. The results demonstrate that SG exhibited pronounced intermittent fluctuations with multiple peaks, whereas CG maintained a relatively stable plateau during the main training phase, reflecting differences in load structure between the two training modalities.

Descriptive analyses of continuous monitoring data showed that SG displayed higher peak V̇O_2_ (2.85 ± 0.45 vs. 2.10 ± 0.30 L/min) and peak heart rate (184 ± 9 vs. 164 ± 10 bpm), as well as a greater proportion of time with RER > 0.95 (34 ± 12% vs. 18 ± 9%) compared with CG (Table 5). These findings suggest more frequent high-intensity metabolic exposure during Sanda sessions, whereas CG showed a more stable moderate-intensity load distribution.

## 4 Discussion

### 4.1 Effects on body composition and cardiorespiratory fitness

This study showed that both structured low-contact Sanda training and MICT improved body composition and cardiorespiratory fitness in young men with obesity, but the adaptive patterns differed between modalities. Body mass and BMI decreased in both groups, whereas body fat percentage showed a larger reduction after MICT. This finding is consistent with previous evidence indicating that aerobic exercise can reduce body mass, fat mass, and central adiposity in adults with overweight or obesity, particularly when sufficient exercise volume is accumulated over time (31, 32). Because body fat reduction is strongly influenced by energy balance and exercise dose, the greater decrease in body fat percentage observed in the MICT group may be partly explained by the prolonged exposure to a stable moderate-intensity workload, which is commonly associated with sustained aerobic metabolism and greater reliance on fat oxidation during exercise (33).

The greater improvements in V̇O_2_peak and VT2 power observed in the Sanda group suggest that structured Sanda training may provide a stronger stimulus for cardiorespiratory adaptation and high-intensity exercise tolerance than steady cycling MICT. This interpretation is consistent with evidence showing that interval-based exercise can induce substantial improvements in aerobic capacity, mitochondrial function, and high-intensity exercise performance through repeated exposure to vigorous physiological stress (34, 35). In the present study, Sanda training involved repeated combinations of footwork, striking actions, defensive movements, and pad-work intervals, which likely produced transient elevations in oxygen uptake, heart rate, and ventilatory demand. These repeated fluctuations may have enhanced the ability to tolerate higher exercise intensities and contributed to the greater improvement in VT2-related capacity.

VT1 improved similarly in both groups, suggesting that both modalities provided sufficient aerobic stimulus to enhance lower-to-moderate-intensity ventilatory regulation. By contrast, the larger improvement in VT2 in the Sanda group may reflect greater exposure to transient high-intensity loads. Ventilatory and lactate threshold-related indices are closely related to endurance performance and the ability to sustain exercise at higher intensities (36, 37). Therefore, the greater VT2 improvement in the Sanda group may indicate enhanced tolerance to metabolic stress during higher-intensity exercise. Nevertheless, because the present study did not verify every high-intensity Sanda segment as being above each participant's VT2, this finding should be interpreted as an adaptation to a HIIT-like intermittent combat-sport modality rather than as evidence that Sanda is equivalent to standardized HIIT.

### 4.2 Effects on substrate oxidation responses

Both training modalities improved substrate oxidation indicators, but the magnitude and direction of adaptation differed. MICT produced larger increases in MFO and FATmax, suggesting a more favorable effect on exercise fat oxidation capacity. This result is physiologically plausible because substrate utilization during exercise is strongly intensity dependent. According to the crossover concept, increasing exercise intensity shifts substrate use progressively from lipid toward carbohydrate oxidation (38). Experimental evidence also shows that carbohydrate utilization rises and fat oxidation decreases as exercise intensity increases (39). Therefore, the stable moderate-intensity workload in MICT may have provided a more sustained stimulus for fat oxidation than the intermittent high-intensity pattern of Sanda training.

The larger increase in FATmax after MICT suggests that participants were able to sustain higher exercise intensities while maintaining relatively high fat oxidation rates after training. This adaptation may be practically relevant for individuals with obesity because exercise performed near FATmax has been proposed as a useful approach for improving fat oxidation and cardiometabolic health (40). Recent meta-analytic evidence also suggests that both HIIT and MICT can improve MFO in adults with overweight or obesity, but moderate-intensity continuous protocols performed near 65%–70% V̇O_2_peak may be particularly effective for optimizing MFO improvements (41). These findings support the interpretation that MICT may be more directly targeted toward improving fat oxidation profiles.

Sanda training also improved MFO and FATmax, although to a lesser extent than MICT. The intermittent high-intensity actions in Sanda likely increased carbohydrate reliance during peaks of effort, as suggested by the higher RER response during training. Such responses may be beneficial for improving V̇O_2_peak and VT2-related exercise capacity, but they may provide less continuous stimulation of fat oxidation pathways. Therefore, the two modalities appear to induce complementary adaptations: MICT may preferentially improve fat oxidation capacity and body fat reduction, whereas Sanda training may be more effective for improving cardiorespiratory fitness and high-intensity exercise tolerance. Whether combining both modalities produces additive or synergistic effects requires confirmation in future studies using a dedicated combined-training design.

### 4.3 Metabolic-load characteristics and interpretation of modality comparison

Continuous respiratory monitoring showed that total session energy expenditure was broadly comparable between the Sanda and MICT groups, but the temporal distribution of metabolic load differed substantially. Sanda training produced repeated short-duration peaks in energy expenditure, V̇O_2_, heart rate, and RER, whereas MICT showed a more stable moderate-intensity plateau. This finding indicates that total energy expenditure alone does not fully describe the physiological stimulus imposed by different exercise modalities. Contemporary training-load frameworks emphasize that external load, internal physiological load, and subjective or biomechanical demands may differ even when total duration or total work appears similar (42–45).

This point is particularly important for interpreting the comparison between Sanda and cycling MICT. Sanda is a weight-bearing, whole-body, multi-joint activity involving technical movements, rapid directional changes, upper- and lower-limb coordination, and intermittent attack–recovery sequences. In contrast, cycling MICT is a non-weight-bearing, cyclic exercise with a more constrained movement pattern. Previous work comparing endurance exercise modalities has shown that cycling and weight-bearing exercise can differ in ventilatory, cardiovascular, neuromuscular, and metabolic responses, even when performed at similar relative intensities (46). Therefore, equal session duration and similar total energy expenditure can standardize the overall exercise dose, but they cannot fully equate biomechanical or systemic physiological load.

The observed adaptive differences are therefore best interpreted as modality-specific responses under equal-duration conditions rather than as the result of fully matched training loads. The greater improvements in V̇O_2_peak and VT2 power after Sanda training may reflect repeated exposure to transient high-intensity metabolic and ventilatory stress, whereas the greater improvements in MFO, FATmax, and body fat percentage after MICT may reflect prolonged time spent in a moderate-intensity range favorable for fat oxidation. This interpretation aligns with the concept that training adaptations are shaped not only by total energy output, but also by intensity distribution, movement pattern, and the interaction between physiological and biomechanical load.

### 4.4 Practical implications

From an applied perspective, the present findings suggest that structured low-contact Sanda training may be considered a practical alternative exercise modality for young men with obesity who aim to improve cardiorespiratory fitness and high-intensity exercise tolerance. Because this intervention was delivered through technical drills, pad work, movement combinations, and controlled attack–recovery exercises rather than full-contact sparring, it may offer a more engaging form of intermittent exercise while reducing the injury concerns typically associated with combat sports. The technical and variable nature of Sanda may also help reduce exercise monotony, which is relevant because enjoyment and affective responses can influence future exercise participation and adherence (47, 48).

However, when the primary goal is body fat reduction or improvement in fat oxidation capacity, MICT may remain a more targeted option. The stable intensity structure of MICT allows participants to spend a longer proportion of the session within an aerobic range that supports sustained fat oxidation. For exercise prescription, this suggests that Sanda and MICT should not be viewed as interchangeable models, but as complementary modalities that may serve different intervention goals. For individuals requiring improved aerobic base and fat oxidation, MICT may be prioritized; for individuals requiring improved cardiorespiratory fitness, movement engagement, and tolerance to higher intensity, structured Sanda training may be useful. Future interventions may consider sequential or combined designs to determine whether these complementary adaptations can be integrated into individualized exercise prescriptions.

### 4.5 Limitations

This study has several limitations. First, the sample size was relatively small and included only young men with obesity, limiting generalizability to women, older adults, or individuals with more severe obesity. In addition, obesity was defined according to the Chinese adult BMI classification rather than the WHO criterion, which may limit direct comparisons with studies conducted in other populations. Second, the present study compared two active exercise interventions and therefore did not include a non-exercise control group; thus, the independent effects of training beyond time or behavioral monitoring cannot be fully isolated. Third, although the two groups were compared under equal-duration conditions and similar total session energy expenditure, these variables do not fully match physiological or biomechanical load across Sanda and cycling. This limitation is particularly relevant because internal and external training loads may diverge across different exercise modalities.

Fourth, the Sanda intervention was structured and low-contact, but the study did not verify each high-intensity segment as being above VT2 or each recovery segment as being below VT1. Therefore, Sanda should be interpreted as a HIIT-like intermittent combat-sport modality rather than a standardized HIIT protocol. Fifth, the duration of obesity was not recorded, which may have influenced baseline fat oxidation capacity and individual responses to exercise training. Sixth, MFO and FATmax were used as indicators of fat oxidation capacity, but metabolic flexibility was not directly assessed using clamp-based, meal-transition, or substrate-switching protocols. Seventh, dietary intake was monitored through guidance and records but was not strictly controlled by providing standardized meals, and quantitative dietary intake was not included in the primary analysis. Because body composition changes are strongly influenced by energy balance, dietary variation cannot be fully excluded as a potential confounder (49–51).

Finally, the statistical analyses did not adjust for baseline values or changes in body mass/body composition using ANCOVA. Therefore, potential baseline or body-size-related confounding cannot be fully excluded. Future studies should include larger samples, both sexes, non-exercise cycling group, more precise dietary assessment, individualized threshold-based training verification, and statistical models that account for baseline values and body-composition changes. Such designs would help clarify the specific contribution of structured Sanda training to metabolic load, substrate oxidation, and cardiorespiratory adaptation in individuals with obesity.

From a practical perspective, the present findings suggest that structured Sanda training may represent an effective alternative to conventional moderate-intensity continuous exercise for improving fat oxidation capacity and cardiorespiratory fitness in young men with obesity.

## 5 Conclusion

Under equal-duration intervention conditions, both 12 weeks of structured Sanda training and MICT on a cycle ergometer improved body composition and cardiometabolic indicators in young men with obesity, but the adaptive profiles differed. Sanda training produced greater improvements in V̇O_2_peak and VT2-related exercise capacity, whereas MICT produced greater improvements in body fat percentage, MFO, and FATmax. Continuous respiratory monitoring showed that Sanda training had a more intermittent metabolic-load pattern with higher peak responses, while MICT showed a more stable moderate-intensity load distribution.

These findings suggest that structured Sanda training may be considered a practical, engaging, low-contact intermittent exercise option for improving cardiorespiratory fitness in young men with obesity, whereas MICT may be more suitable for improving fat oxidation and reducing body fat.

## Data Availability

The raw data supporting the conclusions of this article will be made available by the authors, without undue reservation.
